# Environmentally-Friendly Extraction of Flavonoids from *Cyclocarya paliurus* (Batal.) Iljinskaja Leaves with Deep Eutectic Solvents and Evaluation of Their Antioxidant Activities

**DOI:** 10.3390/molecules23092110

**Published:** 2018-08-22

**Authors:** Xianchao Shang, Jia-Neng Tan, Yongmei Du, Xinmin Liu, Zhongfeng Zhang

**Affiliations:** 1 Tobacco Research Institute of Chinese Academy of Agricultural Sciences, Qingdao 266101, China; sxc3220342@163.com (X.S.); duyongmei@caas.cn (Y.D.); liuxinmin@caas.cn (X.L.); 2 Graduate School of Chinese Academy of Agricultural Sciences, Beijing 100081, China

**Keywords:** *Cyclocarya paliurus*, deep eutectic solvent, extraction, flavonoid, antioxidant activity

## Abstract

Deep eutectic solvents (DESs) are commonly employed as environmentally-friendly solvents in numerous chemical applications owing to their unique physicochemical properties. In this study, a novel and environmentally-friendly extraction method based on ultrasound assisted-deep eutectic solvent extraction (UAE-DES) was investigated for the extraction of flavonoids from *Cyclocarya paliurus* (Batal.) Iljinskaja (*C. paliurus*) leaves, and the antioxidant activities of these flavonoids were evaluated. Nine different DES systems based on either two or three components were tested, and the choline chloride/1,4–butanediol system (1:5 molar ratio) was selected as the optimal system for maximizing the flavonoid extraction yields. Other extraction conditions required to achieve the maximum flavonoid extraction yields from the leaves of *C. paliurus* were as follows: DES water content (*v*/*v*), 30%; extraction time, 30 min; temperature, 60 °C; and solid-liquid ratio, 20 mg/mL. Liquid chromatography-mass spectrometry allowed the detection of five flavonoids in the extract, namely kaempferol-7-*O*-α-l-rhamnoside, kaempferol, quercetin, quercetin-3-*O*-β-d-glucuronide, and kaempferol-3-*O*-β-d-glucuronide. In vitro antioxidant tests revealed that the flavonoid-containing extract exhibited strong DPPH and ABTS radical-scavenging abilities. Results indicate that UAE-DES is a suitable approach for the selective extraction of flavonoids from *C. paliurus* leaves, and DESs can be employed as sustainable extraction media for other bioactive compounds.

## 1. Introduction

*Cyclocarya paliurus* (Batal.) Iljinskaja (*C. paliurus*), which belongs to the genus Cyclocarya Iljinskaja (Juglangdaceae), is a unique and state-protected plant species [1,2]. This plant is widely distributed in the mountains, valleys, and limestone mountains at around 420 to 2500 m above sea level in southern China [3]. Interestingly, the leaves of *C. paliurus* traditionally have been used as a health food [4,5,6], which may be related to its high content of biologically active compounds. Indeed, previous studies have revealed that the leaves of *C. paliurus* contain flavonoids, polysaccharides, internal esters, sterols, saponins, amino acids, and various mineral elements [7,8,9,10]. More specifically, flavonoids have been recognized as the main active compounds in *C. paliurus*, and these compounds exhibit a range of biological activities, which have attracted significant attention over the recent years [7,8,11]. To date, five main flavonoids have been isolated from *C. paliurus* leaves, including kaempferol-7-*O*-α-l-rhamnoside, kaempferol, quercetin, quercetin-3-*O*-β-d-glucuronide, and kaempferol-3-*O*-β-d-glucuronide [12].

Conventional organic solvent-based techniques for the extraction of flavonoids tend to require long extraction times and high extraction temperatures, with the large-scale use of organic solvents being detrimental to the environment. To address these issues, deep eutectic solvents (DESs) have been applied in several areas of chemistry [13,14,15,16,17,18], including various extraction and purification processes. Deep eutectic solvents are environmentally-friendly solvents that exhibit a variety of beneficial properties. For example, they are simple to prepare from cheap starting materials, and their remarkable physicochemical and biodegradable properties render them versatile alternatives to conventional organic solvents [19,20,21]. Besides being biodegradable and non-toxic, DESs reduce the use of petroleum solvent, they have technical performances comparable with those of volatile organic solvents. Moreover, DESs are very suitable to microwave or ultrasound-assisted extraction, because this polar solvents have a higher dielectric constant than organic solvents and can absorb more energy, which would result in better extraction yields and saving energy [22]. Using DESs to extract phenolic compounds from oil can avoid the production of waste water containing phenolic compounds and the use of inorganic acids and alkalis [14]. Deep eutectic solvents consist of simple and naturally occurring compounds with a high safety profile, and they are considered as new environmentally-friendly solvents.

As such, DESs have been employed in the extraction and separation of various bioactive substances such as alkaloids, phenolic compounds, terpenoids, and saponins from plant materials [23,24,25,26,27,28,29,30]. Dai et al. [20] studied the extraction of flavonols from safflower, using different DESs: glucose: choline chloride; lactic acid: glucose and fructose: glucose: sucrose. Hu et al. [31] evaluated the efficiency of 43 DESs in the green extraction of different types of natural compounds from five Chinese herbal medicines. The results indicated that most DESs proved to be efficient solvents for extraction of alkaloids. Duarte et al. [32] present their results of the extraction of phenolic compounds from green coffee beans using different DESs solutions. The results were compared to extractions made using acetone and citric acid solution, it was found that DESs have a high ability to extract phenolic compounds from green coffee beans, which is related with the H-bond interactions that are established between the phenolic compounds and the DESs molecules. All these studies demonstrated that DESs can be used as suitable extraction solvents for selectively and efficiently extracting bioactive compounds from various plant sources.

Conventional techniques to obtain flavonoids, such as heating, boiling, or refluxing, usually require long extraction times and high extraction temperatures, which result in low extraction efficiency due to hydrolysis, ionization, and oxidation during extraction process [33]. Many new extraction technologies have been developed to separate flavonoids from plants, such as ultrasound-assisted extraction (UAE) [25,31], microwave-assisted extraction (MAE) [29], and supercritical extraction [34]. Supercritical extraction (including subcritical water extraction, supercritical fluid extraction, pressurized fluid extraction, and accelerated solvent extraction) was proved to be a suitable technique for isolation of flavonoids [35,36]. Ultrasound-assisted extraction and MAE are more known to be a fast and efficient method for the extraction of flavonoids from plants. Recently, the MAE system was successfully applied for extraction of flavonoids form *C. paliurus* [37]. Herein, we investigate the efficiency of a wide range of DES systems of different compositions for the extraction of flavonoids from *C. paliurus* leaves using the UAE system. To the best of our knowledge, rapid DES-based UAE-assisted extraction of this class of flavonoids has yet to be reported. Optimization of the extraction conditions was also carried out, with a focus on variables such as the water content in the DES, the extraction time and temperature, and the solid–liquid ratio, with the overall aim to maximize the flavonoid yield. In addition, the antioxidant activities of the various flavonoids are determined using the DPPH (1,1-dipheny-2-picrylhydrazyl) and ABTS (2,2′-azinobis-(3-ethylbenzthiazoline-6-sulphonate)) radical-scavenging assays. Overall, our main objective is to establish an efficient and environmentally friendly DES-based method for the extraction of flavonoids from *C. paliurus* leaves. 

## 2. Experimental

### 2.1. Chemicals and Reagents

Kaempferol (K, purity > 99%), quercetin (Q, purity > 99%), kaempferol-7-*O*-α-l-rhamnoside (KRha, purity > 99%), quercetin-3-*O*-β-d-glucuronide (QGlu, purity > 99%), and kaempferol-3-*O*-β-d-glucuronide (KGlu, purity > 99%) standards were obtained from Shanghai Yuan Mu Biotechnology Co., Ltd. (Shanghai, China). Choline chloride, lactic acid, glucose, glycerol, 1,4-butanediol, citric acid, malic acid, urea, xylitol, and malonic acid were purchased from Aladdin Reagent Company (Shanghai, China). 1,1-Dipheny-2-picrylhydrazyl (DPPH) and 2,2′-azinobis-(3-ethylbenzthiazoline-6-sulphonate) (ABTS) were obtained from Shanghai Hao Ocean Biological Technology Co., Ltd. (Shanghai, China). HPLC-grade acetonitrile and methanol were purchased from Shanghai Macklin Biochemical Co., Ltd. (Shanghai, China). Butylated hydroxytoluene (BHT, purity > 98%) and vitamin C (VC, purity > 98%) were obtained from Qingdao Si Yuan Chemical Co., Ltd. (Qingdao, China).

### 2.2. Plant Materials

The leaves of *C. paliurus* were obtained from Suining County, Jiangxi Province, China. The leaf specimen (100 g) was sliced into small pieces, frozen at −80 °C, and freeze-dried over 48 h using a freeze-dryer (Biosafer, Shanghai, China). The freeze-dried leaf portions were ground into a fine powder (60 mesh) using a mill, and stored at 4 °C prior to use.

### 2.3. Preparation of the DESs

The desired DESs were prepared by mixing two or three components at a specific molar ratio under magnetic agitation for 1–4 h at 60–80 °C until a clear and homogeneous liquid was obtained. Nine different DES systems were prepared, as outlined in Table 1.

### 2.4. Optimization of the Extraction Procedure

Different DESs were tested under the same condition using the same protocol (solid to solvent ratio, 20 mg/mL; extraction time, 20 min; extraction temperature, 50 °C). A 70% DES solution in water (*v*/*v*) was employed in the initial screening, precisely weighted 40 mg of leaf sample powder was added to 2 mL of the extraction solvent in a 20 mL test tube. The resulting mixture was subjected to ultrasonication (200 W) at 50 °C for 20 min prior to centrifugation at 9200× *g* for 10 min. The obtained suspension was made up to a volume of 5 mL using either acetonitrile or methanol prior to UHPLC-MS analysis. Each extraction was performed in triplicate, and the extraction yield (*E_y_*) was calculated as follows:

*E_y_* = *C_f_* × *V_s_*/*m_s_* (1)

where *C_f_* is the concentration of each compound present in the DES, *V_s_* is the dilute suspension volume, and *m_s_* is the mass of the sample.

Optimization of the extraction procedure was carried out using the following parameters: type of DES: as indicated in Table 1; DES water content: 10, 20, 30, 40, or 50%; extraction time: 10, 20, 30, 40, or 50 min; extraction temperature: 30, 40, 50, 60, or 70 °C; and solid (sample powder)-liquid (extractant) ratio: 10, 20, 40, 60, or 80 mg/mL.

### 2.5. Liquid Chromatography-Mass Spectrometry (LC-MS) Conditions

Following their extraction from the leaves of *C. paliurus*, the various flavonoids were analyzed by liquid chromatography-mass spectrometry (LC-MS). The mobile phase consisted of methanol (eluent A) and water containing 0.1% formic acid (eluent B). The gradient program was as follows: 0–1 min, 10% B; 1–12.5 min, 10–35% B; 12.5–13 min, 35–45% B; 13–14 min, 45–35% B; 14–15 min, 35–10% B. The flow rate was 0.3 mL/min, the injection volume was 5 μL and the column temperature was maintained at 25 °C. Owing to the high viscosity of the DESs, the eluent was discharged to the waste for the first 3.5 min of each run to protect the ion source.

Liquid chromatography-mass spectrometry was performed in the multiple-reaction monitoring (MRM) and positive electrospray ionization (ESI) modes. The gas temperature was set at 350 °C, and a nitrogen flow rate of 8 L/min was employed along with a nebulizer pressure of 35 psi. All other ESI and MS parameters were optimized individually for each target compound.

### 2.6. Recovery of Extracted Bioactive Compounds from DES Extracts

Brominated-type macroporous resins (SP-270) were tested to recover bioactive compounds from DES extracts. The glass column (25 mm × 600 mm) was wet-packed with 5 g of SP-270 resins. Following extraction under the optimal conditions, the sample solution (2 mL) was loaded onto the column. After washing with deionized water (20 mL), the sample solution was eluted with ethanol (30 mL) at a constant flow rate of 10 mL/min. All ethanol fractions were dried prior to application in the antioxidant activity tests.

### 2.7. DPPH Radical-Scavenging Activity

The radical-scavenging activity of the flavonoid-containing extract was initially examined using the DPPH assay according to the method reported by Pinteus et al. [38,39]. Ten different sample concentrations (i.e., 10–100 μg/mL) were examined. The desired sample (0.1 mL) was added to a solution of DPPH (0.1 mL, 0.2 mM) dissolved in 95% ethanol, then shaken and allowed to stand at 25 °C for 30 min in the absence of light. The absorbance of each sample was measured at 517 nm using a Sunrise absorbance microplate reader (Tecan, Boston, MA, USA), and the radical-scavenging activities were compared to those of known antioxidants, including butylated hydroxytoluene (BHT) and vitamin C (VC). All samples were measured in triplicate. The percentage reduction of the DPPH radical content was expressed as per the following equation:

DPPH radical-scavenging activity (%) = [(*A_blank_* − *A_sample_*)/*A_blank_*] × 100% (2)

where *A_blank_* is the absorbance of the control (i.e., a mixture of all reagents minus the test compound), and *A_sample_* is the absorbance of the test mixture.

Based on the percentage reduction of the DPPH radical content, IC_50_, an index for comparison of the antioxidant activities, was calculated using SPSS 17.0 software. 

### 2.8. ABTS Radical-Scavenging Activity

The radical-scavenging activity of the flavonoid-containing extract was determined using the ABTS assay according to the procedure described by Sun et al. [40,41]. The ABTS radical cation employed herein was prepared by mixing equal volumes of 7-mM ABTS solution and 2.45-mM potassium persulfate solution. The resulting solution was incubated for 12–14 h at 25 °C in the absence of light, and subsequently diluted with ethanol to give an absorbance of 0.70 ± 0.05 units at 734 nm. Ten different sample concentrations were examined here (i.e., 10–100 μg/mL). Thus, the desired sample (0.05 mL) was added to the ABTS solution (0.15 mL) over 30 min in the absence of light and allowed to stand until the absorbance became stable. The absorbance was measured at 734 nm using a Sunrise absorbance microplate reader (Tecan, Boston, MA, USA). Each measurement was carried out in triplicate. Vitamin C and BHT were used as positive controls. The percentage reduction of the ABTS radical content was estimated as follows:

ABTS radical-scavenging activity (%) = [(*A_blank_*− *A_sample_*)/*A_blank_*] × 100% (3)

where *A_blank_* is the absorbance of the control (i.e., a mixture of all reagents minus the test compound) and *A_sample_* is the absorbance of the test mixture.

Based on the percentage reduction in the ABTS content, the IC_50_ value was determined, as described above.

### 2.9. Statistical Analysis

All experiments were performed in triplicate. For data analysis, the SPSS 17.0 software package (SPSS, Chicago, IL, USA) was used to detect statistically significant differences between means [7,11]. Differences associated with *p* values lower than 0.05 were considered significant.

## 3. Results and Discussion

### 3.1. LC-MS Analysis of the Flavonoids

Mass spectrometric analysis of the flavonoids was initially performed over the full scan under both positive and negative ion modes. However, negative mode ESI was selected as the optimal condition since it produced higher responses. In addition, the [M − H]^−^ ion was chosen as the precursor ion for all analytes because of its high relative intensity, and the fragmentor was optimized. Subsequently, the product ions of the various compounds were selected and the collision energy for each ion transition was optimized (Table 2). The fragmentation patterns of the various flavonoids were compared with those reported in the literature previously (Supplemental Figure S1) [4,7,10].

Liquid chromatography-mass spectrometry was employed to determine the concentrations of the extracted flavonoids, and the abbreviations, retention times (t_R_), molecular weights (MW), mass-to-charge (*m*/*z*) values (MS), and MS^2^ fragmentation ions of the flavonoids are listed in Table 2. The five constituent flavonoids were identified as kaempferol (K), kaempferol-7-*O*-α-l-rhamnoside (KRha), quercetin (Q), quercetin-3-*O*-β-d-glucuronide (QGlu), and kaempferol-3-*O*-β-d-glucuronide (KGlu). The calibration curve equations, linearity ranges, limits of detection (LODs), limits of quantitation (LOQs), and coefficients of determination of the standard solutions are listed in Table 3.

### 3.2. Optimization of the DES Extraction Parameters

#### 3.2.1. Influence of the DES Composition

In order to determine the effect of the DES composition on the extraction of the five flavonoids from *C. paliurus* leaves, nine different choline chloride-based DES systems were examined, which contained glucose, citric acid, glycerol, urea, citric acid, glycerol,1,4-butanediol, lactic acid, malonic acid, malic acid, or xylosic alcohol as hydrogen bond donor. The viscosity of the extraction solvent was reduced by the addition of water, with a 70% aqueous solution of DES being employed for the initial screening. The conditions employed for the extraction were as follows: solid–liquid ratio, 20 mg/mL; extraction time, 20 min; extraction temperature, 50 °C. As outlined in Table 4, significant differences in the extractabilities of the various flavonoids were observed by LC-MS for the various DES compositions employed herein.

Clearly, the extraction capacity of the tested DESs varied greatly depending on the type of HBD in DES. Amine- and alcohol-based DESs were superior to those of the sugar- and carboxylic acid-based DESs. In addition, as flavonoids are readily soluble in alkaline solvents, the extraction efficiencies of organic acid-based DESs were lower than those of amine- or alcohol-based DESs. In general, extraction yields of the five flavonoids followed the order QGlu > KRha > KGlu > K > Q. The flavonoid quercetin-3-*O*-β-d-glucuronide was highly extractable in DES-6 (3.59 mg/g), followed by DES-3 (2.85 mg/g), and DES-4 (3.01 mg/g), whereas DES-2 (2.25 mg/g), DES-7 (2.14 mg/g), DES-8 (2.12 mg/g), and DES-9 (2.62 mg/g) demonstrated lower extractability for QGlu. For flavonoid kaempferol-7-*O*-α-l-rhamnoside, DES-6 (3.11 mg/g) led to higher extraction yields, followed by (DES-2, DES-4, DES-7, DES-8, DES-9), lower extraction yields were obtained in sugar-based DES-1 (1.59 mg/g). For flavonoid kaempferol-3-*O*-β-d-glucuronide, DES-6 (0.32 mg/g) showed higher extraction efficiency, while the other DESs shared the same extraction yields except DES-7 (0.13 mg/g). For flavonoids kaempferol and quercetin, DES-2, DES-7, DES-8, DES-9 exhibited higher extraction efficiency than DES-4 and DES-6. The highest quantities of flavonoids were extracted using the solvent combinations of choline chloride:urea (1:1 molar ratio) and choline chloride:1,4-butanediol (1:5 molar ratio). Thus, as a result of the lower cost of 1,4-butanediol, the choline chloride:1,4-butanediol system was selected as the optimal extraction solvent.

We also found that the flavonoid extraction yields decreased upon reducing the choline chloride:1,4-butanediol ratio. Specifically, when this ratio was decreased further to 1:6, the extraction yields decreased (Supplemental Figure S2). In terms of the mechanism of extraction, it has been reported that the alcohol-based hydrogen bond donor and the flavonoid molecules compete for the chloride anion. These species can envelope the chloride anion, resulting in considerable steric hindrance and preventing interactions between the target compounds and the chloride anion. Further decrease in the choline chloride content in the DES would therefore result in insufficient chloride anions being present to interact with the target compounds. However, a low 1,4-butanediol content would result in issues relating to both diffusion and mass transfer, and so DES-6, comprised of choline chloride and 1,4-butanediol in a 1:5 molar ratio, was selected as the optimal solvent for subsequent experiments.

#### 3.2.2. Effect of the DES Water Content

Experiments were conducted to evaluate the effect of the water content in the DES on the flavonoid yields. Thus, DES-6 (choline chloride:1,4-butanediol, 1:5 molar ratio) containing different water contents (i.e., 10, 20, 30, 40, or 50 vol%) was employed for the extraction, which was carried out over 20 min at 50 °C using a solid-liquid ratio of 20 mg/mL.

**Figure 1:**
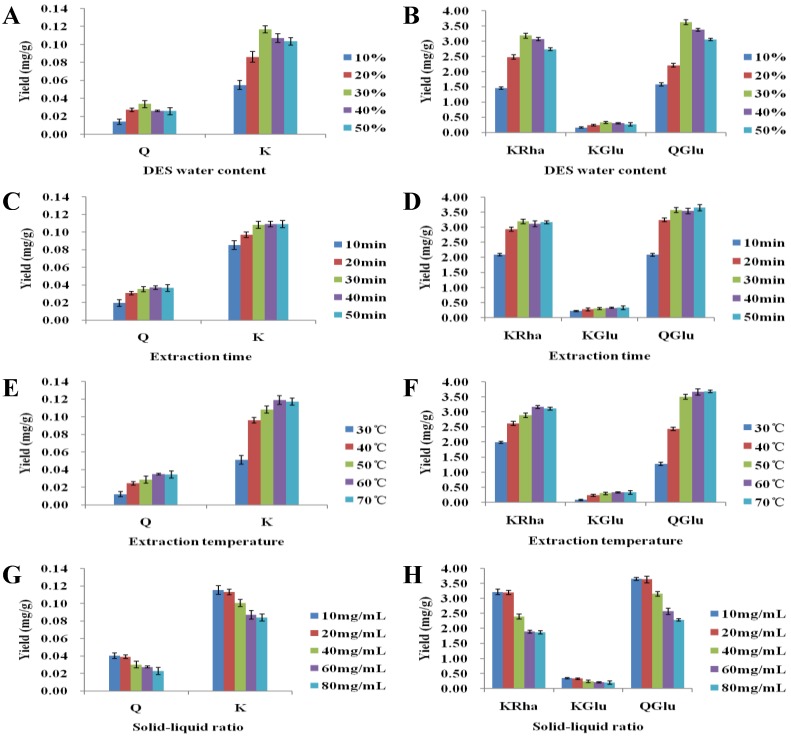
The effect of the water content in the DES, extraction time, temperature, and the solid–liquid ratio on the extraction yield: (**A**,**B**) water content in the DES, (**C**,**D**) extraction time, (**E**,**F**) extraction temperature, and (**G**,**H**) solid–liquid ratio.

The influence of different DES water contents can be seen clearly in Figures. In Figure 1A,B, where it is apparent that the extraction yield depended strongly on the water content. Clearly, upon increasing the water content from 10 to 30%, significant increases in the extraction efficiencies were observed for all five flavonoids. This observation can be most likely attributed to the lower viscosity of the DES, which reduces intermolecular interactions within the solvent and gives rise to increased molecular motion and solubility. However, a further increase in the water content led to decreased extraction efficiencies, and thus water content of 30% was considered as optimal and was employed for all subsequent experiments (extraction yields: Q = 0.034 mg/g, K = 0.117 mg/g, KRha = 3.183 mg/g, KGlu = 0.331 mg/g, and QGlu = 3.628 mg/g). We believe that high water contents may be interfering with the interactions between the flavonoids and the DES systems.

#### 3.2.3. Effect of the Extraction Time

To determine the effect of the extraction time on the obtained flavonoid yields, the samples were extracted over 10, 20, 30, 40, or 50 min, while all other conditions were maintained constant (i.e., DES water content, 30%; extraction temperature, 50 °C; solid–liquid ratio, 20 mg/mL).

The effect of the extraction time on the obtained flavonoid yields is shown in Figure 1C,D. As expected, short extraction times (i.e., 10 or 20 min) resulted in low extraction efficiencies, likely as a result of the limited flavonoid dissolution in the extraction solvent. However, upon increasing the extraction time to 30 min, significant improvements in the extraction efficiencies were observed. Further increasing the extraction time had no remarkable effect, as dissolution was essentially complete after 30 min. Based on these results, an optimal extraction time of 30 min was employed for all subsequent experiments.

#### 3.2.4. Effect of the Extraction Temperature

To determine the optimum extraction temperature for flavonoid extraction, leaf samples were extracted using 30 vol% DES-6 over 20 min at 30, 40, 50, 60, or 70 °C, and the results are outlined in Figure 1E,F. As indicated, upon increasing the extraction temperature from 30 to 60 °C, a clear increase in the flavonoid yields was observed, although no further increases took place at higher temperatures (i.e., 70 °C). An extraction temperature of 60 °C was therefore employed for further experiments.

#### 3.2.5. Effect of the Solid–Liquid Ratio

A range of solid (sample mass)–liquid (extractant volume) ratios were also examined (i.e., 10–80 mg/mL) to determine the optimal conditions for extraction. Interestingly, we found that the flavonoid recoveries tended to increase upon decreasing the ratio from 80 to 20 mg/mL, as shown in Figure 1G,H. This phenomenon was attributed to the excellent extraction capacity of the DES system. When a large sample mass was employed, the extraction capacity of the DES solvent was low, making the extraction slow. By contrast, when a lower sample mass was used, the extraction capacity of the solvent was increased, and a shorter extraction time could be employed. However, at lower solid–liquid ratios (i.e., 10–20 mg/mL), no further increase in the extraction yield was observed. This result can be most likely attributed to complete dissolution of the flavonoids. We therefore determined that a solid–liquid ratio of 20 mg/mL was optimal.

In addition, second-order kinetics for extraction of flavonoids from *C. paliurus* with deep eutectic solvents were also investigated. The results exhibited good linear relationship (r^2^ > 0.98) between the time/yield and time, indicating satisfactory extraction behaviors of deep eutectic solvents from plant system (Supplemental Table S1 and Figure S3).

### 3.3. Evaluation of the Antioxidant Activity

#### 3.3.1. DPPH Radical-Scavenging Activity

**Figure 2:**
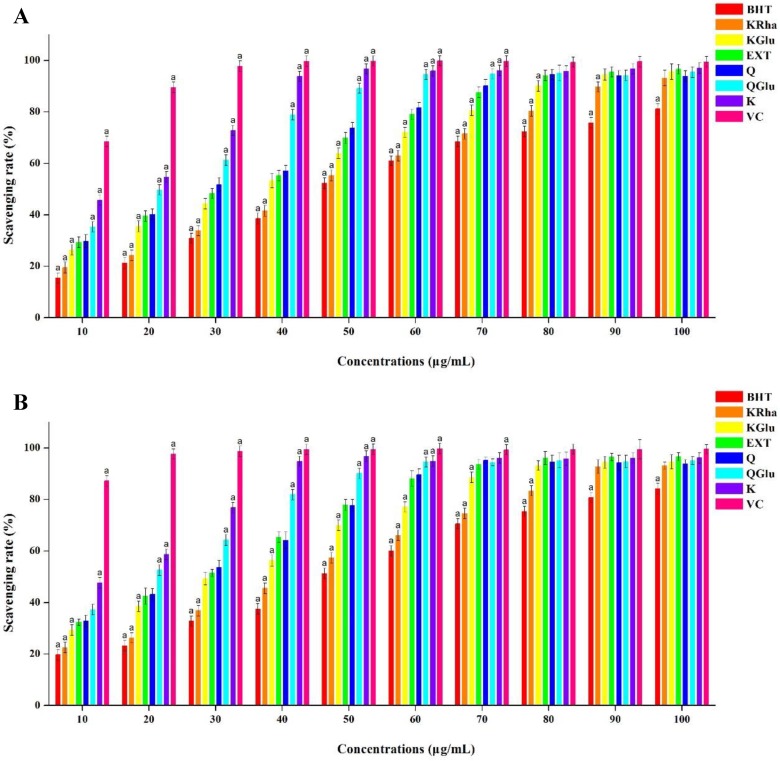
Antioxidant activities of the flavonoids extracted from *C. paliurus* leaves: (**A**) the DPPH radical-scavenging capacity and (**B**) the ABTS radical-scavenging capacity.BHT, butylated hydroxytoluene; KRha, kaempferol-7-*O*-α-l-rhamnoside; KGlu, kaempferol-3-*O*-β-d-glucuronide; Ext, extraction from *C. paliurus*; Q, quercetin; QGlu, quercetin-3-*O*-β-d-glucuronide; K, Kaempferol; VC, vitamin C. Scavenging rates that were significantly different from EXT are indicated with a (*p* < 0.05).

To evaluate the antioxidant (or radical-scavenging) activity of the flavonoids extracted from the *C. paliurus* leaves, we analyzed their DPPH radical-scavenging activity. For a visual contrast, VC and BHT were used as positive controls and the five flavonoids determined were used as comparison, the results are shown in Figure 2A. Indeed, it was clear that the extracted flavonoids exhibited a clear DPPH radical-scavenging activity, with a positive correlation between the radical-scavenging activity and the flavonoid concentration. More specifically, the antioxidant activity of the extract increased from 29.32 ± 1.23% to 96.61 ± 1.01% upon increasing the concentration from 10 to 100 μg/mL. In addition, a higher antioxidant activity was observed for the extracted flavonoids than for BHT at all concentrations, with an IC_50_ value of 25.2 μg/mL. However, a superior radical-scavenging activity was observed for VC, a typical antioxidant, which gave an IC_50_ value of 7.1 μg/mL. With the increase of concentration, especially when the concentration reach 80 μg/mL, the extracts share a similar DPPH radical-scavenging activity with the five flavonoids determined. Statistical analysis results indicate that DPPH radical-scavenging activity of extracts has no difference with quercetin (Q). Based on the above results, it can be concluded that the flavonoids extracted from *C. paliurus* leaves using our optimized DES system exhibit considerable DPPH radical-scavenging activity.

#### 3.3.2. ABTS Radical-Scavenging Activity

We next examined the ABTS radical-scavenging activity of the flavonoid extracts over a range of concentrations. As indicated in Figure 2B, the radical-scavenging activity was enhanced at higher flavonoid concentrations, and a significantly higher activity was obtained than for BHT over the concentration range examined. However, as in the case of the DPPH assay, VC exhibited a superior scavenging activity towards the ABTS radical at concentrations between 10 and 100 μg/mL. Also, ABTS radical-scavenging activity of extracts has no difference with quercetin (Q) at concentrations between 10 and 100 μg/mL. These results confirmed that the flavonoids extracted from *C. paliurus* leaves using our DES system exhibit an excellent antioxidant activity, with an IC_50_ value of 22.4 μg/mL. We can conclude from the results of the DPPH and ABTS assays that our DES system is suitable for the extraction of flavonoids from *C. paliurus* leaves, and that the obtained extracts exhibit excellent antioxidant activities. As such, *C. paliurus* leaves can be considered as a valuable natural source of antioxidants in the human diet, in addition to their potential for use as functional foods or medicines.

## 4. Conclusions

A novel, simple, and environmentally-friendly UAE-DES method was developed for the extraction of five flavonoids from the leaves of *C. paliurus*. We found that choline chloride:1,4-butanediol in a 1:5 molar ratio was the optimal DES system, and this composition was considered to be advantageous because of its facile preparation, in addition to its relatively non-toxic and environmentally-friendly properties. Optimization of the extraction conditions maximized the extraction yields of the five flavonoids (i.e., kaempferol-7-*O*-α-l-rhamnoside, kaempferol, quercetin, quercetin-3-*O*-β-d-glucuronide, and kaempferol-3-*O*-β-d-glucuronide), which were identified by LC-MS. The final optimized conditions were as follows: DES system, choline chloride:1,4-butanediol (1:5 molar ratio); DES water content, 30%; extraction time, 30 min; extraction temperature, 60 °C; and solid-liquid ratio, 20 mg/mL. Under the optimal extraction conditions, the flavonoids obtained from the *C. paliurus* leaves exhibited good in vitro antioxidant activities in both DPPH and ABTS radical-scavenging assays (IC_50_ = 25.2 μg/mL for the DPPH assay, and 22.4 μg/mL for the ABTS assay). We therefore conclude that the developed DES system is a suitable and environmentally-friendly alternative for the extraction of flavonoids and other bioactive compounds from a range of plant species. Further studies are currently under way to elucidate the interactions between the flavonoids compounds and the various DESs.

## Figures and Tables

**Table 1:** Employed DES systems.

DES Code	Component			Molar Ratio
	1	2	3	
DES-1	choline chloride	glucose	-	2:1
DES-2	choline chloride	citric acid	-	1:1
DES-3	choline chloride	glycerol	-	1:1
DES-4	choline chloride	urea	-	1:1
DES-5	choline chloride	citric acid	glycerol	1:1:1
DES-6	choline chloride	1,4-butanediol	-	1:5
DES-7	choline chloride	lactic acid	-	1:1
DES-8	choline chloride	malonic acid	-	1:1
DES-9	choline chloride	malic acid	xylosic alcohol	1:1:1

**Table 2:** Retention times, MWs, *m*/*z* values, and MS^2^ fragmentation ions of the five flavonoids detected in *C. paliurus* leaves.

Flavonoid	t_R_ (min)	MW	*m*/*z* Value	MS^2^ Fragment Ions (*m*/*z*)
QGlu	4.83	478.37	477.10	150.99, 178.95, 300.94
KGlu	5.80	462.36	461.19	133.14, 199.00, 285.05
KRha	6.89	432.09	431.17	227.02, 255.02, 283.89
Q	8.39	302.23	301.00	107.00, 121.01, 151.02
K	10.26	286.23	285.01	117.76, 186.99, 239.00

**Table 3:** Calibration curve equations, linearity ranges, LODs, and LOQs of the standard solutions.

Flavonoid	Regression Equation	Linear Range (μg/g)	Coefficient of Determination (r^2^)	LOD (μg/g)	LOQ (μg/g)
K	y = 33570x + 5715	0.01–0.5	0.9997	0.002	0.007
Q	y = 37130x + 2490	0.01–0.5	0.9994	0.002	0.008
KRha	y = 12705x + 3487	0.5–25	0.9999	0.008	0.025
KGlu	y = 27780x + 32736	0.1–5	0.9995	0.012	0.040
QGlu	y = 49660x + 23127	0.5–25	0.9992	0.006	0.020

**Table 4:** Influence of the DES composition on the extraction yield.

DES System	Extraction Yields of the Target Compounds (mg/g)				
	Q	K	KRha	KGlu	QGlu
DES-1	0.007± 0.001 ^d^	0.018 ± 0.001 ^g^	1.59 ± 0.02 ^f^	0.23 ± 0.01 ^d^	2.60 ± 0.02 ^d^
DES-2	0.021 ± 0.001 ^c^	0.044 ± 0.002 ^d^	2.74 ± 0.02 ^b^	0.25 ± 0.01 ^bc^	2.25 ± 0.01 ^e^
DES-3	0.011 ± 0.000 ^d^	0.027 ± 0.001 ^f^	2.14 ± 0.01 ^e^	0.21 ± 0.00 ^d^	2.85 ± 0.02 ^c^
DES-4	0.010 ± 0.001 ^d^	0.021 ± 0.002 ^fg^	2.73 ± 0.01 ^b^	0.23 ± 0.03 ^c^	3.01 ± 0.05 ^b^
DES-5	0.025 ± 0.001 ^bc^	0.036 ± 0.001 ^e^	2.46 ± 0.01 ^d^	0.25 ± 0.00 ^b^	2.84 ± 0.02 ^c^
DES-6	0.033 ± 0.002 ^a^	0.105 ± 0.004 ^a^	3.11 ± 0.05 ^a^	0.32 ± 0.02 ^a^	3.59 ± 0.04 ^a^
DES-7	0.024 ± 0.001 ^bc^	0.045 ± 0.002 ^d^	2.62 ± 0.03 ^c^	0.13 ± 0.01 ^f^	2.14 ± 0.03 ^ef^
DES-8	0.027 ± 0.001 ^b^	0.067 ± 0.000 ^c^	2.76 ± 0.01 ^b^	0.24 ± 0.01 ^cd^	2.12 ± 0.04 ^f^
DES-9	0.025 ± 0.000 ^bc^	0.094 ± 0.007 ^b^	2.66 ± 0.02 ^c^	0.20 ± 0.01 ^e^	2.62 ± 0.01 ^d^

Data are expressed in the form of mean ± standard deviation (*n* = 3). Values in the same column that do not share the same letter are significantly different (one way ANOVA and Tukey’s test, *p* < 0.05). Extraction conditions: water content, 30%; extraction time, 20 min; extraction temperature, 50 °C; solid-liquid ratio, 20 mg/mL; ultrasonic power, 200 W.

## Supplementary Materials

The following are available online, Figure S1: The UPLC-MS/MS TIC spectrum of the standards (**A**) and target compounds (**B**): kaempferol (K, 10.26 min), quercetin (Q, 8.39), kaempferol-7-*O*-α-l-rhamnoside (KRha, 6.89 min), kaempferol-3-*O*-β-d-glucuronide (KGlu, 5.80), quercetin-3-*O*-β-d-glucuronide (QGlu, 4.83 min); Figure S2: The effects of different ratios of choline chloride/1,4-butanediol on the yields of five flavonoids; Figure S3: Linear fittings of five flavonoids; Table S1: Linear fitting equations of five flavonoids.

## Abbreviations

ABTS	2,2′-azinobis-(3-ethylbenzthiazoline-6-sulphonate)
ANOVA	analysis of variance
BHT	butylated hydroxytoluene
DES	deep eutectic solvent
DPPH	1,1-dipheny-2-picrylhydrazyl
ESI	electrospray ionization
K	kaempferol
KGlu	kaempferol-3-*O*-β-d-glucuronide
KRha	kaempferol-7-*O*-α-l-rhamnoside
IC_50_	concentration of antioxidant where the response is reduced by half
MRM	multiple-reaction monitoring
QGlu	quercetin-3-*O*-β-d-glucuronide
SD	standard deviation
t_R_	retention time
UAE	ultrasound-assisted extraction
UAE-DES	ultrasound assisted-deep eutectic solvent extraction
VC	vitamin C
