# Supplementary material for: Environmentally-Friendly Extraction of Flavonoids from Cyclocarya paliurus (Batal.) Iljinskaja Leaves with Deep Eutectic Solvents and Evaluation of Their Antioxidant Activities
Source: Molecules. 2018 Aug 22;23(9):2110. doi: 10.3390/molecules23092110 (PMC6225260; doi:10.3390/molecules23092110)
Supplement: Supplementary file 1 [file molecules-23-02110-s001.pdf]

Supplemental **Fig. S1.**

Fig. S1.

The UPLC-MS/MS TIC spectrum of the standards (A) and target compounds (B): kaempferol (K, 10.26 min), quercetin (Q, 8.39), kaempferol-7-O-α-L-rhamnoside (KRha, 6.89 min), kaempferol-3-O-β-D-glucuronide (KGlu, 5.80), quercetin-3-O-β-D-glucuronide (QGlu, 4.83 min).

(A)

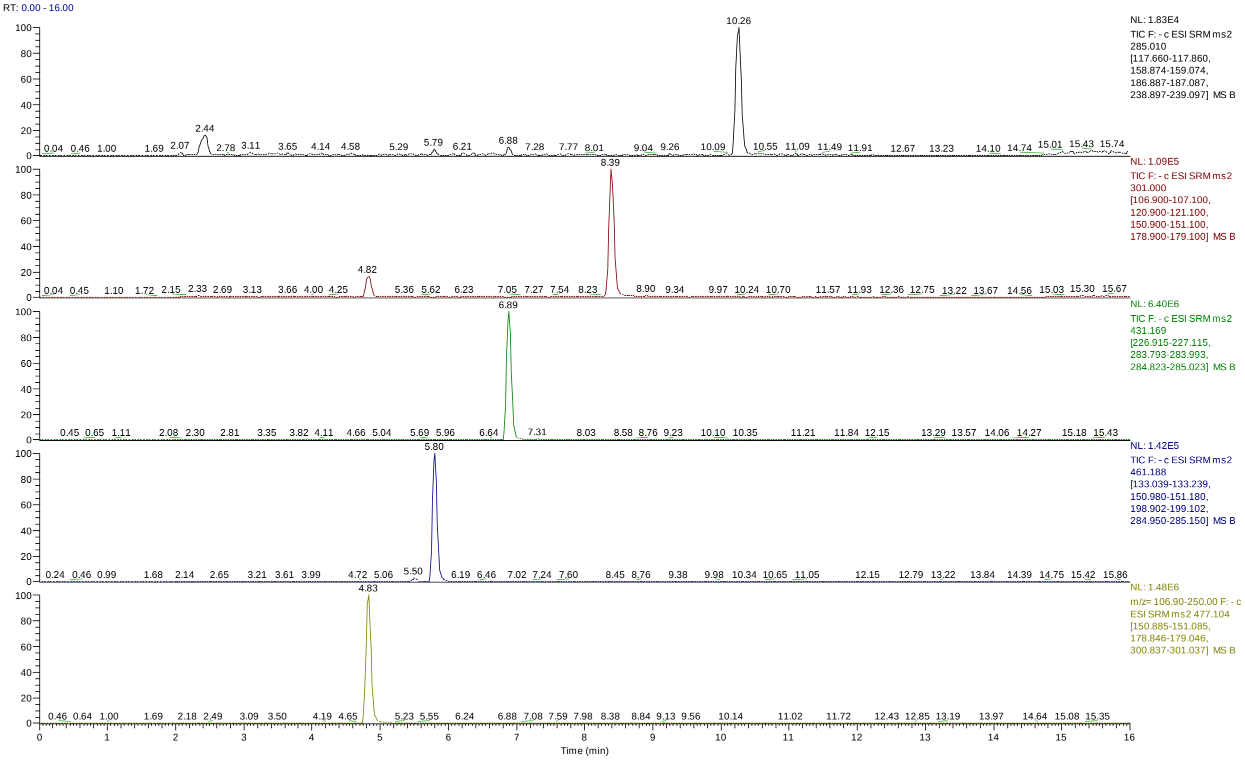

(B)

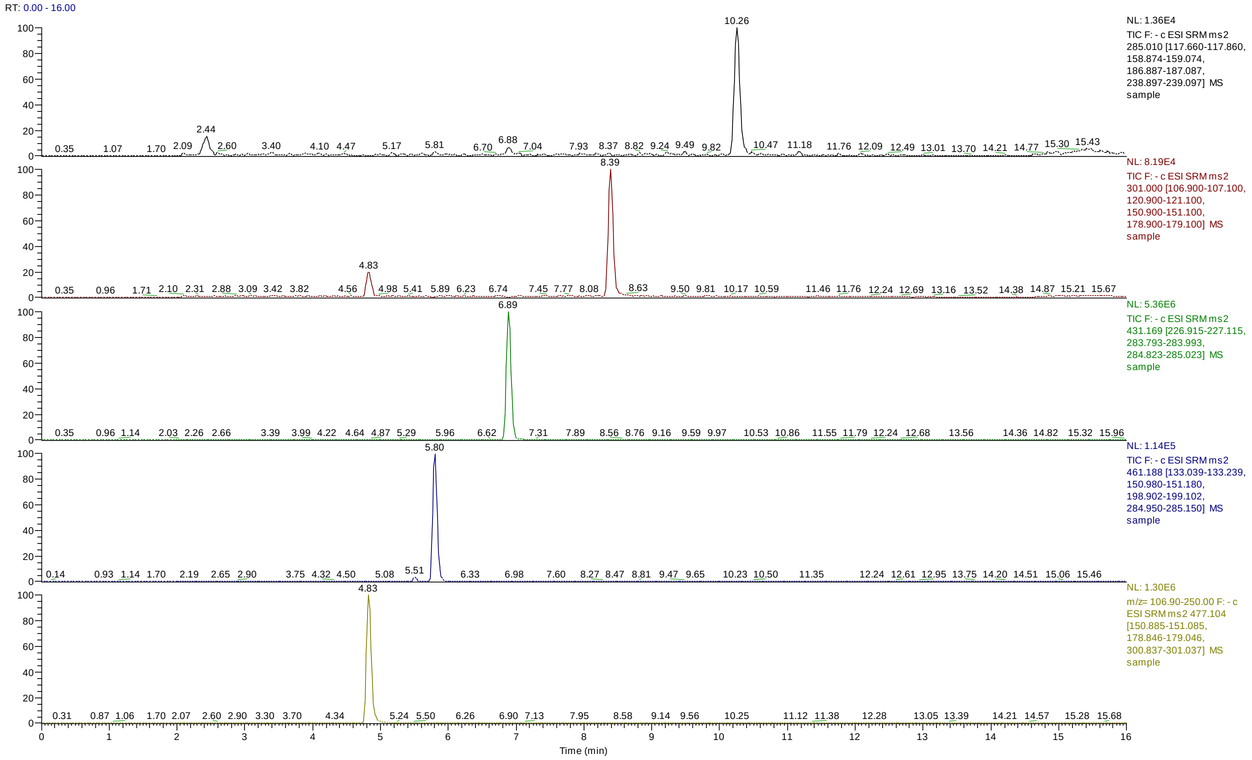

Fig. S2. The effects of different ratios of choline chloride/1,4-butanediol on the yields of five flavonoids.

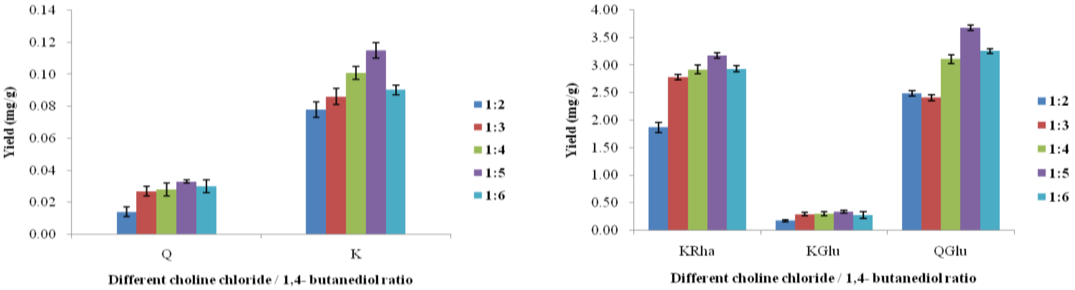

Fig. S3. Linear fittings of five flavonoids.

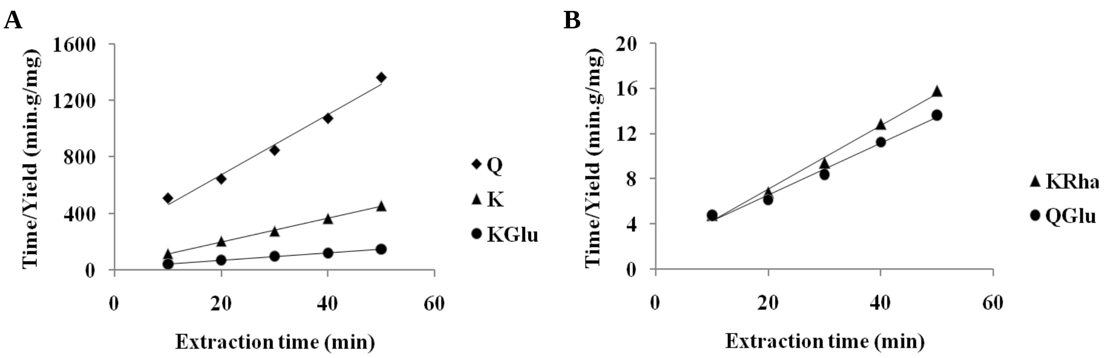

Table S1. Linear fitting equations of five flavonoids.

Flavonoid	Linear fitting equation	Coefficient of determination (r^2)
K	y=8.423x+32.51	0.998
Q	y=21.37x+249.8	0.983
KRha	y=0.280x+1.506	0.991
KGlu	y=2.583x+20.70	0.999
QGlu	y=0.229x+1.987	0.986

The samples were extracted under the optimal conditions. The linear fitting was carried out with the extraction time as the abscissa and the ratio of time to yield as the ordinate.The results in Table S1. and Fig. S3. showed that extraction of flavonoids from *C. paliurus* with deep eutectic solvents tallies with second-order kinetics.
